# Childhood Oral Infections Associate with Adulthood Metabolic Syndrome: A Longitudinal Cohort Study

**DOI:** 10.1177/0022034520929271

**Published:** 2020-06-01

**Authors:** P.J. Pussinen, S. Paju, J. Viikari, A. Salminen, L. Taittonen, T. Laitinen, D. Burgner, M. Kähönen, T. Lehtimäki, N. Hutri-Kähönen, O. Raitakari, M. Juonala

**Affiliations:** 1 Oral and Maxillofacial Diseases, University of Helsinki, Helsinki, Finland; 2 Department of Clinical Physiology and Nuclear Medicine, Turku University Hospital, Turku, Finland; 3 Vaasa Central Hospital, Vaasa, Finland; 4 University of Eastern Finland, Kuopio, Finland; 5 Murdoch Children’s Research Institute, Royal Children’s Hospital, Parkville, Australia; 6 Department of Paediatrics, University of Melbourne, Parkville, Australia; 7 University of Tampere, Tampere, Finland; 8 Finnish Cardiovascular Research Center–Tampere, Faculty of Medicine and Health Technology, Tampere University, Tampere, Finland; 9 Centre for Population Health Research, University of Turku and Turku University Hospital, Turku, Finland; 10 Research Centre of Applied and Preventive Cardiovascular Medicine, University of Turku, Turku, Finland

**Keywords:** caries, gingivitis, inflammation, pediatric dentistry, periodontal disease(s), periodontitis

## Abstract

Chronic oral infection/inflammation is cross-sectionally associated with metabolic syndrome (MetS) in adults, but there are few longitudinal studies and studies on childhood oral infections and adult MetS risk. We investigated whether childhood clinical parameters indicative of oral infection/inflammation were associated with adulthood MetS and its components. A total of 755 children aged 6, 9, and 12 y underwent a clinical oral examination in 1980 as part of the Cardiovascular Risk in Young Finns Study. Oral health measures included bleeding on probing (BOP), periodontal probing pocket depth, caries, fillings, and visible plaque. Metabolic parameters were determined at baseline and during follow-up. MetS was diagnosed (*n* = 588, 77.9%) in the adulthood at 21 y (in 2001), 27 y (in 2007), and 31 y (in 2011) after the oral assessment, when the participants were 27 to 43 y old. Regression analyses were adjusted for childhood age, sex, body mass index, and family income, as well as adulthood smoking and education level. In adulthood, MetS was diagnosed in 11.9% (2001), 18.7% (2007), and 20.7% (2011) of participants at the 3 follow-ups. Childhood caries and fillings were associated with increased risk of adult MetS (risk ratio [95% CI], 1.25 [0.90 to 2.45] and 1.27 [1.02 to 1.99]) and with increased systolic blood pressure (1.78 [1.01 to 4.26] and 2.48 [1.11 to 4.12]) and waist circumference (2.25 [1.02 to 4.99] and 1.56 [1.01 to 3.25]), whereas BOP and visible plaque were associated with plasma glucose (1.97 [1.08 to 3.60] and 1.88 [1.00 to 3.53]). Severity of BOP (*P* = 0.015) and caries (*P* = 0.005) and teeth with plaque (*P* = 0.027) were associated with number of MetS components. No such trends were seen with probing pocket depth. Childhood oral infection/inflammation was associated with adverse metabolic parameters and MetS in adulthood.

## Introduction

Metabolic syndrome (MetS) is a cluster of cardiometabolic measures that increase risk of cardiovascular disease and type 2 diabetes and is defined by at least 3 of the following components: abdominal obesity, hypertension, dysglycemia, and a proatherogenic dyslipidemia (high triglycerides or low high-density lipoprotein [HDL] cholesterol concentration; Grundy et al. 2004). Since immune responses and metabolic pathways are closely linked, failed immune resolution or permanent immune activation may disrupt metabolic homeostasis. Chronic oral infection/inflammation is suggested to contribute to cardiometabolic diseases by direct effects of the dysbiotic oral microbiota or indirectly by resultant inflammation (Pietiäinen et al. 2018).

Periodontitis is associated with all MetS components, including adiposity, insulin resistance, and dyslipidemia, and with MetS in adults per a systematic review (Nibali et al. 2013). The evidence for these cross-sectional associations increases with periodontitis severity (Gomes-Filho et al. 2016) and is more evident in women (Tu et al. 2013). Overall MetS (Cao et al. 2017; Iwasaki et al. 2019) and its components (Ojima et al. 2015) are associated with tooth decay. Among middle-aged nondiabetic participants who had never smoked, MetS was associated with deepened periodontal pockets and caries (Timonen et al. 2010). Apical periodontitis has also been associated with a number of metabolic disorders (Sasaki et al. 2016), and missing teeth (or edentulousness), the endpoint of periodontitis and caries, have been robustly associated with MetS (Hyvärinen et al. 2015).

There are few studies investigating the association of oral and metabolic health in children, and findings are inconsistent (Manohar et al. 2020). Pathologic periodontal pockets were associated with diastolic blood pressure in obese adolescents (Zeigler et al. 2015), and adolescents with caries had more cardiovascular risk factors than those without caries (Larsson et al. 1995). Gingivitis in children and adolescents correlated with the number of MetS components (Kâ et al. 2013; Lee et al. 2015).

In the few longitudinal data available, caries experience at the age of 15 y predicted central obesity 3 y later (Li et al. 2017), and deferred dental care during adolescence was associated with increased body mass index (BMI) in early adulthood (Oreskovic et al. 2017). In our previous study of the Cardiovascular Risk in Young Finns Study cohort, increasing evidence of childhood oral infection/inflammation (i.e., caries, fillings, bleeding on probing [BOP], and increased periodontal probing pocket depth [PPD]) was associated with child- and adulthood cumulative cardiovascular disease risk factors (Pussinen et al. 2019).

Given the lack of prospective studies of oral infection/inflammation and MetS, we aimed to investigate the association of caries and periodontal diseases in childhood and MetS and its components in adulthood. Our hypothesis was that caries and periodontal diseases in childhood associate with unfavorable metabolic features.

## Methods

### Population

Description of the ongoing Cardiovascular Risk in Young Finns Study, including analyses of attrition to show the representativeness of the cohort, has been published previously (Raitakari et al. 2008). The current analysis included 755 participants who had a baseline evaluation during childhood in 1980, including a clinical oral examination (Pussinen et al. 2019). The age groups were 6 y (*n* = 213, 28.2%), 9 y (*n* = 275, 36.4%), and 12 y (*n* = 267, 35.4%). Clinical cardiometabolic follow-up was performed in adulthood (years 2001, 2007, 2011). Participants represent a random subpopulation of their age groups of the whole cohort, but in the follow-up examinations, the proportion of females was higher (40.1% vs. 53.9%, *P* = 0.002; Pussinen et al. 2019). The prevalence of oral health parameters (caries, fillings, BOP, and PPD) was similar between those with and without the clinical follow-up (Appendix Table 1). The study complies with the Declaration of Helsinki, and the Ethics Committee of the Hospital District of Southwest Finland approved the research protocol. Informed consent was obtained from the participants or their parents. Reporting of the study follows the STROBE guidelines.

### Oral Examinations

Oral examinations were performed in 1980 when the 755 participating children were 6, 9, and 12 y old (Pussinen et al. 2019). Children were examined at university dental schools in 5 cities in Finland (Helsinki, Kuopio, Tampere, Turku, and Oulu). Oral hygiene data (brushing frequency per day) were obtained by questionnaire. In the oral examination, we recorded the number of teeth (deciduous and permanent), the presence of visible plaque, and present or previous (i.e., treated) dental infections (dental caries and dental fillings) and periodontal diseases (gingival BOP and periodontal PPD). The presence of caries and fillings was recorded from 5 surfaces of the tooth (mesial, buccal, distal, lingual, and occlusal). Visible plaque was recorded as present or absent at 4 areas of dentition: maxillary and mandibular incisors as well as maxillary and mandibular premolars and molars. Periodontal probing was performed on 2 sites (maxillary teeth: mesial and buccal; mandibular teeth: mesial and lingual) of 6 index teeth (maxilla: right first molar, left central incisor, left first premolar; mandible: left first molar, right central incisor, right first premolar). PPD of the gingival sulcus was categorized as no pocketing, 0 to 1.9 mm; slight gingival deepening and shallow periodontal pockets, 2 to 5.9 mm; and deep periodontal pockets, >6 mm. BOP was observed after probing and recorded as present or absent. Proportions of sites with increased PPD or BOP were calculated.

### Clinical and Biochemical Assessment

Clinical and biochemical assessments have been extensively described (Porkka 1997; Juonala 2004; Raitakari et al. 2008; see Appendix Methods).

### MetS Diagnosis

Adulthood MetS was defined per widely accepted international criteria (Alberti et al. 2009) and childhood MetS according to the modified National Cholesterol Education Program (Expert Panel 2001; see Appendix Methods). The number of participants assessed at least once for MetS was 588 (77.9%). In 2001, 2007, and 2011, 472 (62.5%), 476 (63.0%), and 441 (58.4%) participants were assessed for MetS, respectively (Appendix Table 2).

### Statistical Analyses

The weighted terms of linear trends between measured parameters and oral findings were analyzed by analysis of variance, followed by Fisher’s least significant difference for post hoc comparisons. Differences between groups were analyzed via *t* test and chi-square test for continuous and categorical measures, respectively. Correlations were analyzed by the Pearson correlation test.

The associations were examined by using linear regression and Poisson regression models, which were minimally adjusted for age and sex (model 1). If the associations resulted in *P* values ≤0.1, the models were further adjusted for childhood BMI, family income, and interaction terms between caries and periodontal findings, as well as adulthood smoking status (never/ever) and education level (basic, occupational, and academic; model 2).

The oral parameters were used as continuous or categorical variables. For continuous variables, caries parameters were combined in DMFT (decayed, missing, and filled teeth), whereas the percentage of sites with either BOP or increased PPD (≥2 mm) was summed up to represent periodontal diseases. For categories, registered parameters were classified in 2 ways: present or absent. To combine parameters representing caries, 3 categories were structured: 1) neither caries nor fillings, 2) only untreated caries, and 3) only treated caries. To combine parameters representing gingival inflammation, 3 groups were created: 1) no BOP, 2) BOP <30%, and 3) BOP ≥30%. The 30% cutoff was the mean value of participants with BOP.

## Results

Caries, fillings, BOP, increased PPD, and visible plaque were common and registered in 53.4% to 86.4% of participants (Appendix Fig.). Only slight gingival deepening and shallow periodontal pockets (≥2 mm) were found. The number of sites with BOP was positively correlated with the number of sites with increased PPD (*r* = 0.226, *P* < 0.001) and teeth with visible plaque (*r* = 0.364, *P* < 0.001).

### Childhood MetS and Childhood Oral Infections

Frequencies of childhood MetS and its components according to childhood oral infections are presented in Appendix Table 3. From a population of 567 participants with complete data, 70 (12.3%) had MetS (*n* = 29 boys [41.4%], *n* = 41 girls [58.6%], *P* = 0.311). MetS or the number of MetS components did not differ between groups with different oral findings. However, caries and fillings were more common in participants with high systolic blood pressure, high BMI, and low HDL cholesterol. BOP and increased PPD were more common in participants with high BMI and high glucose, respectively.

### Adulthood MetS and Childhood Oral Infections

The characteristics of the population stratified by adulthood MetS are presented in Table 1 (each year separately in Appendix Table 4). MetS was diagnosed in 56 (11.9%), 89 (18.7%), and 91 (20.7%) participants in 2001, 2007, and 2011, respectively. During the whole follow-up, 153 (26.0%) participants were diagnosed at least once with MetS, which was more prevalent in males than females. In addition to the metabolic parameters, the participants with or without adulthood MetS differed significantly regarding age, childhood family income, and adulthood smoking status and education level.

**Table 1.** Characteristics of the Population Stratified by the Presence of MetS in Adulthood.

	Mean (SD)				
Childhood Characteristic	No (*n* = 433)		Yes (*n* = 153)		*P* Value^a^
Age, y	7.8 (2.0)		8.4 (1.9)		**0.001**
Systolic blood pressure, mm Hg	109 (9)		113 (10)		**<0.001**
Diastolic blood pressure, mm Hg	67 (9)		69 (10)		**0.034**
BMI, kg/m^2^	16.5 (2.1)		17.3 (2.2)		**<0.001**
Cholesterol, mmol/L	5.4 (0.87)		5.4 (0.93)		0.538
HDL cholesterol, mmol/L	1.64 (0.30)		1.59 (0.33)		0.131
Triglycerides, mmol/L	0.58 (0.26)		0.65 (0.28)		**0.003**
LDL cholesterol, mmol/L	3.47 (0.79)		3.54 (0.85)		0.409
Family income (among 8 classes)	5.51 (1.72)		4.89 (1.73)		**<0.001**
	*n* (%)				*P* Value^b^
Sex: male	183 (42.4)		87 (56.1)		**0.003**
Daily toothbrushing					0.761
No	34 (8.0)		10 (6.7)		
Once	238 (55.9)		88 (59.1)		
At least twice	154 (36.2)		51 (34.2)		
Childhood Oral Parameter^c^	Presence, *n* (%)	Extent, Mean (SD)	Presence, *n* (%)	Extent, Mean (SD)	
Only untreated caries^d^	21 (4.8)	1.00	4 (2.6)	1.00	
Caries^d^	369 (85.0)	5.16 (3.09)	140 (90.3)	5.65 (3.00)	
Fillings^d^	347 (80.2)	5.41 (3.00)	136 (87.7)	5.79 (2.94)	
Bleeding on probing^e^	273 (62.9)	28.9 (16.9)	105 (67.7)	34.3 (21.7)	
Increased probing pocket depth^e^	223 (53.7)	26.1 (17.3)	80 (52.6)	26.7 (18.9)	
Visible plaque^f^	324 (75.0)	2.56 (1.10)	119 (76.8)	2.74 (1.12)	
Adulthood Characteristic	*n* (%)				*P* Value^b^
Ever smoker	110 (25.3)		53 (34.2)		**0.035**
Education					**0.004**
No	5 (1.4)		0 (0)		
Basic	12 (2.8)		5 (3.2)		
Occupational	253 (58.3)		114 (73.5)		
Academic	163 (37.6)		36 (27.2)		

BMI, body mass index; HDL, high-density lipoprotein; LDL, low-density lipoprotein.

a *t* test.

b Chi-square test.

c The childhood oral parameters of participants having at least 1 clinical examination in the adulthood during 2001, 2007, or 2011 (*n* = 588).

d If caries or fillings were present, their extent is presented as mean number of teeth affected among the whole dentation.

e If bleeding or increased pocket depth (≥ 2 mm) was found on probing, extent is presented as mean proportions of sites registered from 2 surfaces of 6 teeth.

f If plaque was present, its extent is presented as mean number of teeth with plaque registered from 4 index teeth.

The habits of toothbrushing did not differ between those with and without MetS (Table 1). The presence of caries or fillings in deciduous and permanent teeth did not differ between participants with and without MetS (Appendix Table 5) and was further analyzed together.

**Figure 1.**
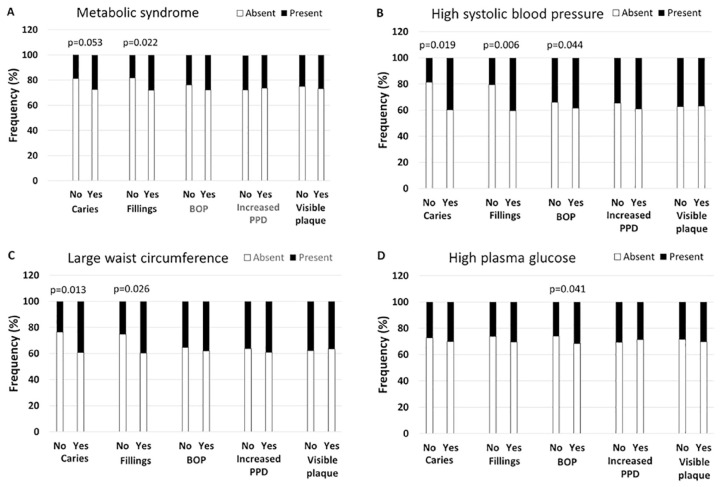
Presence of childhood oral infection/inflammation and adulthood metabolic syndrome (MetS). A total of 755 children had a clinical oral examination in 1980, and 4 signs of oral infections were registered: caries, fillings, bleeding on probing (BOP), and increased probing pocket depth (PPD). The presence of these symptoms is shown among participants diagnosed in adulthood with MetS (**A**) or one its components: high systolic blood pressure (**B**), large waist circumference (**C**), and high glucose (**D**). Significant *P* values are shown for differences between the groups after adjusting for age and sex. The whole data for all years and all MetS components are presented in Appendix Table 3.

The frequencies and mean values of affected teeth or proportion of sites is presented in Table 1 according to the MetS diagnosis (each year separately in Appendix Table 6). Childhood caries, fillings, BOP, and visible plaque were more common in participants with MetS in adulthood than in those without (Fig. 1A). In the presence of these oral findings, the risk ratios (95% CI) for adulthood MetS were 1.25 (0.90 to 2.45) for caries, 1.27 (1.02 to 1.99) for fillings, 1.13 (0.75 to 1.71) for BOP, and 1.21 (0.87 to 1.86) for plaque (Table 2).

**Table 2.** Association of Presence of Childhood Oral Parameters with the Presence of Adulthood MetS and Its Components in Any Examination Point.

	MetS Component^a^											
	High Systolic Blood Pressure		Large Waist		Low HDL		High Glucose		High Triglycerides		MetS^a^	
Parameter:^b^ Model	RR (95% CI)	*P* Value	RR (95% CI)	*P* Value	RR (95% CI)	*P* Value	RR (95% CI)	*P* Value	RR (95% CI)	*P* Value	RR (95% CI)	*P* Value
Caries												
1	**1.99(1.11 to 3.56)**	**0.019**	**2.02(1.16 to 3.50)**	**0.013**	1.19(0.82 to 1.72)	0.356	0.81(0.52 to 1.26)	0.351	1.32(0.83 to 2.08)	0.236	1.56(0.98 to 2.55)	0.053
2	**1.78(1.01 to 4.26)**	**0.048**	**2.25(1.02 to 4.99)**	**0.046**							1.25(0.90 to 2.45)	0.070
Fillings												
1	**2.17(1.25 to 3.76)**	**0.006**	**1.75(1.07 to 2.86)**	**0.026**	1.06(0.75 to 1.50)	0.746	0.98(0.63 to 1.53)	0.940	1.08(0.71 to 1.63)	0.731	**1.41(1.08 to 2.19)**	**0.022**
2	**2.48(1.11 to 4.12)**	**0.010**	**1.56(1.01 to 3.25)**	**0.048**							**1.27(1.02 to 1.99)**	**0.044**
Bleeding on probing												
1	**1.33(1.00 to 1.85)**	**0.044**	1.17(0.85 to 1.60)	0.341	1.01(0.77 to 1.33)	0.940	**1.41(1.02 to 2.01)**	**0.041**	1.02(0.75 to 1.40)	0.888	1.16(0.90 to 1.55)	0.077
2	1.23(0.82 to 2.24)	0.190					**1.97(1.08 to 3.60)**	**0.028**			1.13(0.75 to 1.71)	0.464
Increased PPD												
1	0.99(0.73 to 1.34)	0.933	0.99(0.73 to 1.34)	0.993	0.93(0.71 to 1.21)	0.589	0.79(0.57 to 1.08)	0.143	1.01(0.75 to 1.37)	0.933	0.82(0.62 to 1.09)	0.167
Visible plaque												
1	1.26(0.78 to 1.78)	0.170	1.24(0.88 to 2.00)	0.218	1.19(0.89 to 1.59)	0.242	1.37(0.94 to 1.99)	0.106	1.36(0.96 to 1.94)	0.053	1.30(0.94 to 1.78)	0.101
2							**1.88(1.00 to 3.53)**	**0.050**	1.62(0.91 to 2.88)	0.101	1.21(0.87 to 1.86)	0.222

Model 1 adjusted for age and sex. Model 2 was calculated if the *P* value in model 1 was ≤0.1. It was additionally adjusted for childhood body mass index and family income, adulthood smoking (ever) and socioeconomic status (education), and interactions terms between caries and periodontal parameters.

HDL, high-density lipoprotein; MetS, metabolic syndrome; PPD, probing pocket depth; RR, risk ratio.

a MetS or its components diagnosed in any adulthood examination point (2001, 2007, or 2011, *n* = 588).

b Oral infection/inflammation as presence of caries, fillings, bleeding on probing, increased probing pocket depth, or visible plaque.

### MetS Components and the Presence of Childhood Oral Infections

Among MetS components, childhood caries, fillings, and BOP were more frequent among participants with high systolic blood pressure in adulthood (Fig. 1B). In fully adjusted regression models, the RRs (95% CI) for high systolic blood pressure were 1.78 (1.01 to 4.26), 2.48 (1.11 to 4.12), and 1.23 (0.82 to 2.24) in the presence of childhood caries, fillings, and BOP, respectively. Caries and fillings were more often found in participants with large waists in adulthood than in those without (Fig. 1C), producing RRs of 2.25 (1.02 to 4.99) and 1.56 (1.01 to 3.53) in the fully adjusted models. Additionally, BOP (RR, 1.97 [1.08 to 3.60]) was more common among participants with high plasma glucose (Fig. 1D) and visible plaque (RR, 1.88 [1.00 to 3.53]) among those with high triglyceride concentrations.

### MetS Components and the Extent of Childhood Oral Infections

In linear regression models, only the proportion of sites with BOP and the number of teeth with visible plaque were significantly associated with the number of positive MetS components (Table 3). The largest effect sizes were observed with the cumulative sum of all MetS components, with β values (*P* value) of 0.123 (0.026) for BOP and 0.120 (0.027) for visible plaque. The associations of oral findings and separate MetS components are presented in Appendix Table 7. Extent of caries was consistently associated with diastolic blood pressure with an increase of 0.8 to 3.7 mm Hg for every affected tooth. Additionally, the association between the extent of periodontal disease and either systolic or diastolic blood pressure level was consistent (increase of 0.3 to 1.5 mm Hg for any additional affected site). The associations between periodontal findings and BMI or waist circumference were of borderline significance or inconsistent.

**Table 3.** Association of the Extent of Childhood Oral Parameters with the Number of Positive Metabolic Syndrome Components in Adulthood in Linear Regression Models.

	Year of MetS Assessment							
	2001		2007		2011		Cumulative^a^	
Parameter: Model	Beta (*P* Value)	*R* ^2^	Beta (*P* Value)	*R* ^2^	Beta (*P* Value)	*R* ^2^	Beta (*P* Value)	*R* ^2^
Teeth with caries^b^								
1	0.019 (0.674)	0.000	0.005 (0.921)	0.001	0.020 (0.669)	0.002	0.013 (0.812)	0.000
Teeth with fillings^b^								
1	0.020 (0.680)	0.001	0.035 (0.458)	0.005	0.067 (0.180)	0.012	0.017 (0.779)	0.005
Proportion of bleeding on probing^c^								
1	**0.085 (0.049)**	**0.008**	0.050 (0.277)	0.002	**0.124 (0.010)**	**0.012**	**0.163 (0.004)**	**0.023**
2	0.065 (0.098)				**0.094 (0.047)**		**0.123 (0.026)**	
Proportion of probing pocket depth^c^								
1	0.046 (0.328)	0.002	0.057 (0.219)	0.003	0.014 (0.777)	0.000	0.004 (0.942)	0.000
Teeth with visible plaque^b^								
1	0.061 (0.189)	0.005	**0.096 (0.035)**	**0.010**	**0.116 (0.013)**	**0.012**	**0.179 (0.001)**	**0.034**
2			0.075 (0.086)		0.073 (0.100)		**0.120 (0.027)**	

In 2001, 2007, and 2011, 472 (62.5%), 476 (63.0%), and 441 (58.4%) participants were assessed for metabolic syndrome (MetS), respectively. Model 1 adjusted for age and sex. Model 2 was calculated if the *P* value in model 1 was ≤0.1. It was additionally adjusted for childhood body mass index and family income, as well as adulthood smoking (ever) and socioeconomic status (education). *R*^2^ values are reported from corresponding unadjusted simple linear regressions with fitting models. Statistically significant results are highlighted in bold.

a All adulthood examination years combined (2001, 2007, and 2011, *n* = 588).

b Number of teeth with caries, fillings, or visible plaque.

c Percentage of sites with bleeding or increased pocket depth on probing.

**Figure 2.**
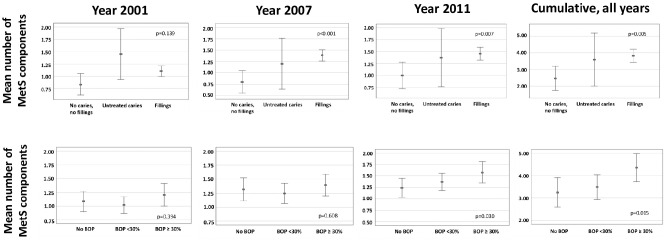
Number of positive metabolic syndrome (MetS) components in adulthood according to the presence of oral infection/inflammation in childhood. The mean number of positive MetS components (95% CI) is shown for adulthood in years 2001 (*n* = 461), 2007 (*n* = 465), and 2011 (*n* = 426) as well as for all years combined (*n* = 588). Participants were divided into 3 groups according to the presence of caries and gingival inflammation: 1) no caries or fillings, 2) only untreated caries present, and 3) only treated caries present and 1) no bleeding on probing (BOP), 2) BOP <30%, and 3) BOP ≥30%. *P* values are shown for weighted linear trend in analysis of variance.

The number of positive MetS components was analyzed according to caries and gingival inflammation (Fig. 2). Signs of caries demonstrated a linear trend with the number of MetS components in 2007 (*P* < 0.001), in 2011 (*P* = 0.007), and cumulatively in all examination years (*P* = 0.005). Similar trends were seen with gingival inflammation in 2011 (*P* = 0.030) and cumulatively in all examinations (*P* = 0.015).

## Discussion

In this longitudinal cohort of children participating in the Cardiovascular Risk in Young Finns Study, we found that childhood caries and periodontal disease were associated with several adverse metabolic parameters and MetS in adulthood. Oral infection/inflammation was most strikingly associated with blood pressure, waist circumference, glucose, and the number of positive MetS components, whereas the associations were less evident for lipid parameters.

Childhood caries and fillings were associated with MetS at any of the 3 examination time points, with an overall 26% increased risk. When compared with earlier, markedly larger studies conducted among adults, the risk was of similar magnitude as in a Finnish cross-sectional study of nondiabetic participants (Timonen et al. 2010), higher than that reported among Chinese (Cao et al. 2017), but lower than that observed among Japanese males (Ojima et al. 2015). The association is plausible, since caries and metabolic conditions are strongly modulated by dietary behavior. The association between oral health status and cardiovascular disorders may be mediated via lifestyle choices such as diet.

The presence of childhood parameters related to periodontal status—that is, BOP and PPD—was not associated with the MetS diagnosis in adulthood, but in linear regression models, the proportion of BOP and the extent of visible plaque were associated with the number of positive MetS components independent of age, sex, smoking, childhood BMI, or childhood and adulthood socioeconomic status. The association between the presence of periodontitis and MetS in adults was explored in a systematic review (Nibali et al. 2013). The causal direction is unclear, since most of the studies are cross-sectional. In one study, the presence of periodontal pockets was associated with an appearance of positive MetS components during the 4-y follow-up (Morita et al. 2010). None of the children in the present study had periodontitis, but childhood gingival inflammation was present at the same time as increased diastolic blood pressure, BMI, and glucose (Pussinen et al. 2019) and preceded increased systolic blood pressure in adulthood.

Early-life socioeconomic disadvantage has adverse effects on adult health in general and adult oral health in particular (Schwendicke et al. 2015). This may be due to socially patterned behaviors learned in early life, such as smoking, poor diet, neglected oral hygiene, and infrequent dental care. As a sign of inadequate oral hygiene habits in the present study, visible plaque was present in 75.3% of the participants, and only 32.6% reported brushing teeth twice daily. In addition, smoking was very common, reported in 23% of the adult participants. In Finland, children learn toothbrushing and oral hygiene techniques in school as part of health education. Additionally, accessibility to dental treatment is not dependent on the family income, since all children, adolescents, and university students are entitled to free oral health care. Nevertheless, family income during childhood and education level in adulthood were considered in the multivariate analyses of the present study as indicators of socioeconomic position. This may influence the exposure and outcome through numerous pathways and has been shown to associate strongly with adulthood MetS (Puolakka et al. 2016).

Childhood caries was associated with large waist circumference in adulthood. Similar trends were seen in periodontal diseases, although the results were not fully consistent at all follow-up appointments. However, these results are in keeping with 2 longitudinal studies in adolescents and young adults (Li et al. 2017; Oreskovic et al. 2017) and meta-analyses (Martens et al. 2017; Manohar et al. 2019). Key determinants of these associations are dietary risk factors, especially carbohydrate intake, but the mechanisms seem to differ between caries and periodontitis (Chapple et al 2017). In caries, local glycemia leads to disturbances with acid production and cariogenic biofilm components, whereas in periodontitis, systemic hyperglycemia and oxidative stress trigger advanced glycation end products leading to increased/heightened inflammatory responses and metabolic dysfunction (Chapple et al. 2017; Detzen et al. 2019).

Oral infection/inflammation in childhood was associated with diastolic and systolic blood pressure in adulthood, independent of childhood BMI. In our longitudinal analysis, caries and fillings were associated with 78% and 148% increased risk for high systolic blood pressure, and the extent of periodontal findings had a linear association with blood pressure values in adulthood independent of childhood BMI. Epidemiologic data and evidence from clinical trials data suggest that excessive dietary sugar intake results in increased blood pressure in children and young adults (Chapple et al. 2017). In addition, endodontic pathology was particularly associated with hypertension in a recent large study (Messing et al. 2019). In a meta-analysis, moderate to severe periodontitis was associated with hypertension (Muñoz Aquilera et al. 2020), and intensive periodontal treatment decreased blood pressure (Czesnikiewicz-Guzik et al. 2019). Furthermore, a mendelian randomization analysis demonstrated a significant association between periodontitis-linked genotype and blood pressure phenotype (Czesnikiewicz-Guzik et al. 2019). Our results further support a role of oral infection/inflammation in development of hypertension and emphasize the importance of oral care in hypertension prevention already from early in life.

Childhood BOP was associated with a substantially increased risk for having high glucose levels in adulthood. The bidirectional association between periodontitis and diabetes is well known: diabetes increases the risk of periodontitis and disturbs periodontal treatment. Moreover, periodontitis and missing teeth associate with the risk of incident diabetes (Liljestrand et al. 2015; Winning et al. 2017), and their role in prediabetes has been widely investigated (Kocher et al. 2018). High fasting plasma glucose from childhood to midadulthood is associated with impaired fasting glucose and diabetes in adulthood (Campbell et al. 2018). This raises a question whether childhood gingival inflammation is associated with diabetes risk in adulthood and could be considered an early risk indicator.

In the present study, the presence of caries and the extent of gingival inflammation were associated with MetS components. The number of cardiovascular disease risk factors is higher in adolescents with caries than in those without (Larsson et al. 1995), and the number of MetS components correlates with gingivitis in adolescents and children (Franchini et al. 2011; Kâ et al. 2013; Lee et al. 2015). No association between childhood oral inflammation and adult lipid level was observed in the present study, although periodontitis has been associated with reduction of HDL functionality and HDL cholesterol and with elevation of low-density lipoprotein cholesterol and triglyceride concentrations in adults (Pussinen et al. 2004; Pietiäinen et al. 2018).

The main strengths of the study include its unique design and long follow-up with versatile metabolic measurements. We, however, also acknowledge some limitations. Neither clinical attachment level was measured nor radiographic examination performed, and periodontal probing was performed on only 6 index teeth. Increased PPD was not associated with MetS or its components. Increased PPD was often registered with BOP, and these 2 parameters were weakly correlated. In the whole population, however, increased PPD was a frequent (53.9%) albeit mild finding, since only slight gingival deepening and shallow periodontal pockets were observed, and no deep periodontal pockets were found. Increased PPD may in part represent pseudopockets associated with newly erupting teeth, thereby decreasing reliability of this registered parameter in children. Gingival inflammation precedes periodontitis but does not necessarily lead to it in nonsusceptible individuals. Unfortunately, oral health, visits to dentist, treatments, oral hygiene habits, or use of fluoride has not been recorded since the baseline in 1980, and it remains unclear whether these longitudinal associations are independent of adulthood dental status. In addition, information on diet or lifestyle interventions during the follow-up are missing, and the follow-up of all 755 examined children was not complete. Since multiple comparisons were made, the results must be interpreted with caution. The limited number of participants in each clinical examination may have caused inconsistences in the results.

We found convincing evidence of associations between childhood caries and adulthood MetS—specifically, the number of MetS components, hypertension, and abdominal obesity. Gingival inflammation in childhood was associated with the number of MetS components, hypertension, and high glucose levels, whereas its association with BMI or abdominal obesity was not consistent throughout the follow-up. Some of these associations were apparent already in childhood. In conclusion, our longitudinal study suggests that chronic oral infection/inflammation in childhood is associated with child- and adulthood metabolic dysfunction and with MetS in adults. Further studies including more detailed assessment of oral health and mechanistic studies may highlight targets for intervention in childhood to reduce cardiometabolic risk in adults.

## Author Contributions

P.J. Pussinen, contributed to conception, data analysis, and interpretation, drafted the manuscript; S. Paju, A. Salminen, contributed to data acquisition and interpretation, critically revised the manuscript; J. Viikari, O. Raitakari, M. Juonala, contributed to conception, design, data acquisition, and interpretation, critically revised the manuscript; L. Taittonen, T. Laitinen, contributed to design, data acquisition, and interpretation, critically revised the manuscript; D. Burgner, M. Kähönen, T. Lehtimäki, N. Hutri-Kähönen, contributed to design and data acquisition, critically revised the manuscript. All authors gave final approval and agree to be accountable for all aspects of the work.

## Supplemental Material

DS_10.1177_0022034520929271 – Supplemental material for Childhood Oral Infections Associate with Adulthood Metabolic Syndrome: A Longitudinal Cohort Study Supplemental material, DS_10.1177_0022034520929271 for Childhood Oral Infections Associate with Adulthood Metabolic Syndrome: A Longitudinal Cohort Study by P.J. Pussinen, S. Paju, J. Viikari, A. Salminen, L. Taittonen, T. Laitinen, D. Burgner, M. Kähönen, T. Lehtimäki, N. Hutri-Kähönen, O. Raitakari and M. Juonala in Journal of Dental Research
