# Supplementary material for: Childhood Oral Infections Associate with Adulthood Metabolic Syndrome: A Longitudinal Cohort Study
Source: J Dent Res. 2020 Jun 1;99(10):1165–73. doi: 10.1177/0022034520929271 (PMC7443963; doi:10.1177/0022034520929271)

Supplementary material:

Childhood oral infections associate with adulthood metabolic syndrome:

A longitudinal cohort study

Pussinen PJ[1], Paju S[1], Viikari J[2], Salminen A[1], Taittonen L[3], Laitinen T[4], Burgner D[5, 6], Kähönen M[7], Lehtimäki T[8], Hutri-Kähönen N[7], Raitakari O[2, 9, 10], Juonala M[2]

Methods: Clinical and biochemical assessment

Metabolic syndrome diagnosis

Supplementary table 1. Frequencies of oral infections in those participating or not in adulthood clinical follow-up.

Supplementary Table 2. Number of participants in the adulthood follow-ups.

Supplementary table 3. Frequencies of oral infections according to childhood MetS and its components.

Supplementary table 4. Characteristics of the population.

Supplementary table 5. Frequencies of caries and fillings in deciduous and permanent teeth and MetS.

Supplementary Table 6. Presence of metabolic syndrome and its components in the follow-up according to the presence of childhood oral parameters.

Supplementary table 7. Association of childhood oral parameters with the metabolic parameters in linear regression models.

Supplementary figure. Frequencies of oral infections in children with or without MetS in adulthood.

Clinical and biochemical assessment

Height, weight, and waist/hip circumference were measured at all examinations using standardized protocols (Raitakari 2008). Baseline blood pressure was measured by a mercury sphygmomanometer, in follow-up studies a random zero mercury sphygmomanometer was used. Blood pressure was measured in the sitting position after a 5-minute rest and the average of three measurements was used. Venous blood samples were collected after a 12-hour fast. Triglyceride, total cholesterol, HDL cholesterol and glucose concentrations were determined as previously described and the levels were corrected for the changes in the methodology (Porkka 1997; Juonala 2004).

MetS diagnosis

Adulthood MetS was defined using widely accepted international criteria (Alberti et al. 2009), and diagnosed by the presence of at least three of the following five components: 1) waist circumference ≥102 cm for males and ≥88 cm for females; 2) triglycerides ≥1.7 mmol/L (≥150 mg/dL) or specific treatment for hypertriglyceridemia; 3) HDL cholesterol <1.0 mmol/L (<40 mg/dL) in males or <1.3 mmol/L (<50 mg/dL) in females or lipid-lowering medication; 4) blood pressure ≥130/85 mmHg or treatment for hypertension; and 5) fasting plasma glucose ≥5.6 mmol/L (≥100 mg/dL) or specific drug treatment of elevated glucose. The number of participants assessed at least once for MetS was 588 (77.9%). In 2001, 2007, and 2011, 472 (62.5%), 476 (63.0%), and 441 (58.4%) participants were assessed for MetS, respectively.

In the present sub-population we calculated the prevalence according to the modified National Cholesterol Education Program (Expert Panel 2001). A participant was categorized as having MetS if he or she had any 3 of the following 5 components: BMI or waist circumference ≥75th percentile; systolic or diastolic blood pressure ≥75th percentile; HDL-cholesterol ≤25th percentile; triglycerides ≥75th percentile; or insulin/glucose ≥75th percentile.

References:

Alberti KG, Eckel RH, Grundy SM, Zimmet PZ, Cleeman JI, Donato KA, Fruchart JC, James WP, Loria CM, Smith SC Jr. Harmonizing the metabolic syndrome: a joint interim statement of the International Diabetes Federation Task Force on Epidemiology and Prevention; National Heart, Lung, and Blood Institute; American Heart Association; World Heart Federation; International Atherosclerosis Society; and International Association for the Study of Obesity. 2009. Circulation. 120(16):1640-1645.

Expert Panel on Detection, Evaluation, and Treatment of High Blood Cholesterol in Adults. 2001. Executive Summary of The Third Report of The National Cholesterol Education Program (NCEP) Expert Panel on Detection, Evaluation, And Treatment of High Blood Cholesterol In Adults (Adult Treatment Panel III). JAMA. 285(19):2486-2497.

Juonala M, Viikari JS, Hutri-Kähönen N, Pietikäinen M, Jokinen E, Taittonen L, Marniemi J, Rönnemaa T, Raitakari OT. 2004. The 21-year follow-up of the Cardiovascular Risk in Young Finns Study: risk factor levels, secular trends and east-west difference. J Intern Med. 255(4):457-468.

Porkka KV, Raitakari OT, Leino A et al. 1997. Trends in serum lipid levels during 1980–1992 in children and young adults. The Cardiovascular Risk in Young Finns Study. Am J Epidemiol. 146:64–77.

Raitakari OT, Juonala M, Rönnemaa T, Keltikangas-Järvinen L, Räsänen L, Pietikäinen M, Hutri-Kähönen N, Taittonen L, Jokinen E, Marniemi J, et al. 2008. Cohort profile: the cardiovascular risk in Young Finns Study. Int J Epidemiol. 37(6):1220-1226.

Supplementary table 1. Frequencies of oral infections in those participating or not in adulthood clinical follow-up.

Clinical examination in the follow-up	Presence of childhood oral parameters in 1980														
	Caries			Fillings			Bleeding on probing			Increased probing pocket depth			Visible plaque		
	Frequency (%)		P[2]	Frequency (%)		P[2]	Frequency (%)		P[2]	Frequency (%)		P[2]	Frequency (%)		P[2]
	no	yes		no	yes		no	yes		no	yes		no	yes	
**No, n=167**	19 (19.2)	148 (22.6)	0.452	29 (21.6)	138 (22.2)	0.883	53 (20.2)	114 (23.3)	0.341	71 (21.2)	89 (22.8)	0.611	19 (19.2)	148 (22.6)	0.452
**Yes, n=588**	80 (80.8)	508 (774)		105 (78.4)	483 (77.8)		210 (79.8)	378 (76.8)		264 (78.8)	302 (77.2)		80 (80.8)	508 (77.4)	

[2] Chi-square test.

Supplementary Table 2. Number of participants in the adulthood follow-ups.

		Number (%)[1] of participants examined			
	**Year**	**2011**	**2007**	**2001**	
**Adulthood**	**2001**	349 (46.2)	384 (50.9)	472 (62.5)	
	**2007**	376 (49.8)	476 (63.0)		
	**2011**	441 (58.4)			
		**Number (%)[1] of participants examined**			
	**Not**	**Once**	**Twice**	**Three times**	**At least once**
	167 (22.1)	95 (12.6)	185 (24.5)	308 (40.8)	588 (77.9)

[1] Number and percentage from the whole population, n=755.

Supplementary table 3. Frequencies of oral infections according to childhood MetS and its components.

MetS components in 1980[1]		Presence of childhood oral parameters in 1980														
		Caries			Fillings			Bleeding on probing			Increased probing pocket depth			Visible plaque		
		Frequency (%)		P[2]	Frequency (%)		P[2]	Frequency (%)		P[2]	Frequency (%)		P[2]	Frequency (%)		P[2]
		no	yes		no	yes		no	yes		no	yes		no	yes	
**High systolic blood pressure**		15 (8.0)	172 (92.0)	**0.020**	20 (10.7)	167 (89.3)	**0.004**	73 (39.0)	114 (61.0)	0.155	82 (44.3)	103 (55.7)	0.581	54 (28.9)	133 (71.1)	0.120
**High BMI**		12 (6.3)	177 (93.7)	**0.001**	14 (7.4)	175 (92.6)	**<0.001**	53 (28.0)	136 (72.0)	**0.023**	85 (45.0)	104 (55.0)	0.692	50 (26.7)	137 (73.3)	0.471
**Low HDL**		32 (17.3)	153 (82.7)	0.056	43 (23.2)	142 (76.8)	**0.023**	69 (37.3)	116 (62.7)	0.419	81 (45.0)	99 (55.0)	0.742	40 (21.6)	145 (78.4)	0.263
**High glucose[3]**		20 (27.0)	140 (28.2)	0.830	26 (26.8)	134 (28.3)	0.761	61 (31.0)	99 (26.5)	0.264	51 (20.9)	98 (32.5)	**0.003**	39 (27.5)	120 (28.2)	0.871
**High TG**		18 (9.8)	166 (90.2)	0.118	27 (14.7)	157 (85.3)	0.218	57 (31.0)	127 (69.0)	0.206	84 (46.2)	98 (53.8)	0.976	42 (22.8)	142 (77.2)	0.497
**Number of childhood MetS components without glucose[4]**	**0 n=309**	46 (14.9)	263 (85.1)	0.197	62 (20.1)	247 (79.9)	0.094	114 (36.9)	195 (63.1)	0.523	136 (47.4)	151 (52.6)	0.799	78 (25.3)	230 (74.7)	0.462
	**1 n=226**	34 (15.0)	192 (85.0)		45 (19.9)	181 (80.1)		73 (32.3)	153 (67.7)		102 (46.2)	119 (53.8)		47 (21.0)	177 (79.0)	
	**2 n=146**	13 (8.9)	133 (91.1)		18 (12.3)	128 (87.7)		52 (35.6)	94 (64.4)		63 (43.8)	81 (56.3)		43 (29.5)	103 (70.5)	
	**3 n=51**	4 (7.8)	47 (92.2)		6 (11.8)	45 (88.2)		14 (27.5)	37 (72.5)		21 (41.2)	30 (58.8)		12 (23.5)	39 (76.5)	
	**4 n=18**	1 (5.6)	17 (94.4)		1 (5.6)	17 (94.4)		8 (27.5)	10 (55.6)		10 (55.6)	8 (44.4)		4 (22.2)	14 (77.8)	
**Number of childhood MetS components with glucose (year 1986)[5]**	**0 n=176**	26 (14.8)	150 (85.2)	0.729	35 (19.9)	141 (80.1)	0.361	61 (34.7)	115 (65.3)	0.995	83 (50.3)	82 (49.7)	0.402	44 (25.0)	132 (75.0)	0.189
	**1 n=182**	27 (14.8)	155 (85.2)		36 (19.8)	146 (80.2)		63 (34.6)	119 (65.4)		76 (44.2)	96 (55.8)		41 (22.8)	139 (77.2)	
	**2 n=139**	14 (10.1)	125 (89.9)		17 (12.2)	122 (87.8)		49 (35.3)	90 (64.7)		55 (40.1)	82 (59.9)		44 (31.7)	95 (68.3)	
	**3 n=49**	5 (10.2)	44 (89.8)		7 (14.3)	42 (85.7)		16 (32.7)	33 (67.3)		20 (41.7)	28 (58.3)		7 (14.3)	42 (85.7)	

	**4 n=19**	2 (10.5)	17 (89.5)		2 (10.5)	17 (89.5)		6 (31.6)	13 (68.4)		9 (47.4)	10 (52.6)		5 (26.3)	14 (73.7)	
	**5 n=2**	0	2 (100)		0	2 (100)		1 (50.0)	1 (50.0)		0	2 (100)		0	2(100)	
**MetS (≥ 3 components)**	**No n=497**	67 (13.5)	430 (86.5)	0.418	88 (17.7)	409 (82.3)	0.313	173 (34.8)	324 (65.2)	0.748	214 (45.1)	260 (54.9)	0.621	129 (26.1)	366 (73.9)	0.107
	**Yes n=70**	7 (10.0)	63 (90.0)		9 (12.9)	61 (87.1)		23 (32.9)	47 (67.1)		29 (42.0)	40 (58.0)		12 (17.1)	58 (82.9)	

[1] Presence of a positive MetS component is defined as belonging to the highest quartile (the lowest quartile of HDL cholesterol). [2] Chi-square test. [3] Plasma glucose concentration determined in 1986; [4] N=750; [5] N=567.

Supplementary table 4. Characteristics of the population.

Characteristic		Adulthood examination year								
		Year 2001			Year 2007			Year 2011		
		MetS in adulthood								
		No (n=414)	Yes (n=56)		No (n=386)	Yes (n=89)		No (n=349)	Yes (n=91)	
		N (%)		P[1]	N (%)		P[1]	N (%)		P[1]
Sex (males)		185 (44.5)	33 (58.9)	**0.042**	168 (43.4)	50 (56.2)	**0.029**	145 (41.5)	50 (54.3)	**0.028**
Current smoker		102 (25.0)	15 (26.8)	0.773	82 (23.9)	23 (29.1)	0.334	57 (17.5)	16 (19.3)	0.712
Education	Basic	35 (8.5)	5 (8.9)	0.712	19 (4.9)	4 (4.5)	**0.049**	7 (2.1)	4 (4.8)	**0.013**
	Occupational	269 (65.0)	39 (69.6)		238 (61.8)	67 (75.3)		200 (61.3)	62 (74.7)	
	Academic	110 (26.6)	12 (21.4)		128 (33.2)	18 (20.2)		119 (36.5)	17 (20.5)	
**Characteristic in 1980**		**Mean (SD)**		**P[2]**	**Mean (SD)**		**P[2]**	**Mean (SD)**		**P[2]**
Age (years)		8.0 (2.0)	8.5 (1.8)	0.057	7.8 (2.1)	8.5 (1.9)	**0.010**	7.8 (2.0)	8.6 (1.9)	**0.001**
Systolic blood pressure (mmHg)		109 (9)	113 (10)	**0.003**	109 (9)	113 (10)	**<0.001**	109 (9)	113 (9)	**0.001**
Diastolic blood pressure (mmHg)		67 (9)	69 (10)	0.102	67 (9)	68 (10)	0.655	67 (9)	69 (10)	0.074
BMI (kg/m^2)		16.6 (2.0)	17.5 (2.3)	**0.003**	16.6 (2.1)	17.3 (2.3)	**0.003**	16.5 (2.2)	17.3 (2.2)	**0.004**
Cholesterol (mmol/l)		5.4 (0.88)	5.6 (0.85)	0.239	5.4 (0.86)	5.4 (0.98)	0.745	5.4 (0.91)	5.3 (0.90)	0.483
HDL cholesterol (mmol/l)		1.64 (0.31)	1.56 (0.31)	0.064	1.65 (0.30)	1.56 (0.31)	**0.010**	1.64 (0.31)	1.59 (0.30)	0.234
Triglycerides (mmol/l)		0.58 (0.25)	0.67 (0.26)	**0.023**	0.58 (0.25)	0.68 (0.27)	**0.002**	0.58 (0.27)	0.68 (0.28)	**0.003**
LDL cholesterol (mmol/l)		3.49 (0.81)	3.68 (0.78)	0.093	3.49 (0.78)	3.58 (0.88)	0.370	3.49 (0.82)	3.42 (0.83)	0.450
Family income (among 8 classes)		5.33 (1.74)	4.82 (1.76)	**0.040**	5.45 (1.74)	4.91 (1.66)	**0.009**	5.52 (1.70)	4.89 (1.71)	**0.002**

[1] Chi-square test; [2] t-test.

Supplementary table 5. Frequencies of caries and fillings in deciduous and permanent teeth and MetS.

Year of MetS assessment, presence of MetS		Caries in childhood oral examination											
		Untreated caries, n (%)						Treated caries, n (%)					
		Deciduous teeth			Permanent teeth			Deciduous teeth			Permanent teeth		
		With	Without	P[1]	With	Without	P[1]	With	Without	P[1]	With	Without	P[1]
**2001**	**No**	138 (89.6)	278 (87.4)		129 (88.4)	287 (88.0)		221 (87.7)	195 (88.6)		241 (85.8)	175 (91.6)	
	**Yes**	16 (10.4)	40 (12.6)	0.965	17 (11.6)	39 (12.0)	0.656	31 (12.3)	25 (11.4)	0.331	40 (14.2)	16 (8.4)	0.372
**2007**	**No**	138 (87.9)	249 (78.1)		117 (84.2)	270 (80.1)		210 (81.1)	177 (81.6)		226 (78.7)	161 (85.2)	
	**Yes**	19 (12.1)	70 (21.9)	0.052	22 (15.8)	67 (19.9)	0.108	49 (18.9)	40 (18.4)	0.338	61 (21.3)	28 (14.8)	0.972
**2011**	**No**	119 (86.2)	230 (75.9)		103 (78.6)	246 (79.4)		197 (82.8)	152 (74.9)		196 (74.5)	153 (86.0)	
	**Yes**	19 (13.8)	73 (24.1)	0.126	69 (24.6)	28 (21.4)	0.603	41 (17.2)	51 (25.1)	0.299	67 (25.5)	25 (14.0)	0.246
**Any**	**No**	158 (79.0)	276 (71.0)		133 (73.9)	301 (73.6)		244 (74.8)	190 (72.2)		240 (69.2)	194 (80.2)	
	**Yes**	42 (21.0)	112 (29.0)	0.287	47 (26.1)	108 (26.4)	0.434	82 (25.2)	72 (27.8)	0.696	107 (30.8)	48 (19.8)	0.208

p-values are adjusted for age and sex.

Supplementary Table 6. Presence of metabolic syndrome and its components in the follow-up according to the presence of childhood oral parameters.

Assessment year of MetS and its components		Presence of childhood oral parameters in 1980														
		Caries			Fillings			Bleeding on probing			Increased probing pocket depth			Visible plaque		
		Frequency (%)		P^2	Frequency (%)		P^2	Frequency (%)		P^2	Frequency (%)		P^2	Frequency (%)		P^2
		no	yes		no	yes		no	yes		no	yes		no	yes	
**High systolic blood pressure**	**2001**	**4.5**	**22.3**	**0.012**	**8.0**	**22.5**	**0.032**	17.9	21.0	NS	21.9	18.8	NS	21.2	19.6	NS
	**2007**	**11.3**	**28.9**	**0.018**	**9.2**	**30.1**	**0.002**	26.4	26.4	NS	25.2	28.7	NS	32.3	24.4	NS
	**2011**	**11.6**	**27.5**	**0.048**	**12.9**	**27.9**	**0.010**	**18.6**	**28.9**	**0.025**	22.3	27.2	NS	**19.5**	**27.1**	**0.050**
	**Any**	**17.0**	**40.9**	**0.019**	**17.2**	**41.9**	**0.008**	**29.8**	**41.4**	**0.044**	35.6	39.1	NS	30.1	40.0	NS
**Large waist**	**2001**	9.0	18.0	NS	11.5	17.9	NS	14.6	17.9	NS	19.7	15.1	NS	13.7	17.6	NS
	**2007**	**14.5**	**28.9**	**0.035**	**16.5**	**29.1**	**0.049**	22.0	29.6	NS	26.4	28.2	NS	**20.0**	**28.9**	**0.001**
	**2011**	**21.7**	**38.2**	**0.049**	**22.4**	**38.7**	**0.048**	33.5	36.9	NS	36.8	35.9	NS	38.1	34.5	NS
	**Any**	**21.6**	**43.2**	**0.012**	**24.2**	**43.4**	**0.017**	34.5	42.9	NS	41.4	40.6	NS	34.8	41.4	NS
**Low HDL**	**2001**	36.8	35.5	NS	40.9	34.5	NS	36.6	35.1	NS	38.7	33.5	NS	30.7	37.3	NS
	**2007**	**18.1**	**33.8**	**0.003**	25.3	32.8	NS	28.5	33.2	NS	31.2	32.3	NS	**24.2**	**34.3**	**0.045**
	**2011**	33.3	33.2	NS	35.3	32.8	NS	32.3	33.8	NS	**39.6**	**28.0**	**0.014**	**25.4**	**35.9**	**0.048**
	**Any**	49.1	50.5	NS	53.8	49.5	NS	50.8	50.0	NS	53.2	46.7	NS	44.2	52.5	NS
**High glucose**	**2001**	8.8	10.6	NS	10.2	10.4	NS	9.1	11.1	NS	11.1	9.7	NS	7.9	11.3	NS
	**2007**	15.3	20.7	NS	14.8	21.0	NS	19.0	20.4	NS	**22.8**	**18.0**	**0.037**	20.3	19.8	NS
	**2011**	20.3	26.5	NS	20.0	26.8	NS	**20.4**	**28.6**	**0.037**	27.6	24.4	NS	21.2	27.2	NS
	**Any**	32.7	33.3	NS	30.0	33.9	NS	**25.8**	**37.7**	**0.021**	36.7	31.0	NS	26.3	36.0	NS
**High TG**	**2001**	23.5	24.3	NS	27.3	23.6	NS	24.6	24.1	NS	27.2	22.6	NS	23.6	24.6	NS
	**2007**	20.8	25.2	NS	21.6	25.2	NS	**21.2**	**25.3**	**0.019**	22.3	25.7	NS	23.4	24.8	NS
	**2011**	14.5	21.3	NS	17.6	20.9	NS	22.8	18.8	NS	20.3	18.6	NS	18.6	20.9	NS
	**Any**	30.9	40.4	NS	36.4	39.5	NS	37.5	39.7	NS	37.4	40.3	NS	**30.5**	**42.3**	**0.046**
**MetS**	**2001**	6.2	12.8	NS	8.2	12.7	NS	12.6	11.6	NS	15.5	19.7	NS	11.5	12.1	NS
	**2007**	11.6	19.9	NS	**11.0**	**20.3**	**0.049**	19.9	18.0	NS	18.2	19.6	NS	19.5	18.5	NS
	**2011**	17.9	21.4	NS	16.9	21.8	NS	18.8	22.1	NS	23.4	19.5	NS	21.4	20.7	NS
	**Any**	**26.8**	**43.7**	**0.048**	**28.4**	**43.9**	**0.049**	35.7	44.4	NS	45.8	39.5	NS	34.3	43.9	NS

[1] Year in adulthood when MetS or its component was diagnosed; [2] p-values adjusted for age and sex. Statistically significant results are highlighted in bold.
MetS = metabolic syndrome; NS = not significant

Supplementary table 7. Association of childhood oral parameters with the metabolic parameters in linear regression models.

Period	Year		DMFT		Percentage of sites with BOP or increased PPD	
			Beta, p	R^2	Beta, p	R^2
**Childhood**	**1980**	BMI	0.041, 0.290		0.034, 0.415	
		Systolic blood pressure	0.022, 0.575		0.005, 0.893	
		Diastolic blood pressure	**0.080, 0.026**	0.014	**0.129, 0.001**	0.015
		HDL-cholesterol	-0.039, 0.287		-0.063, 0.106	
		Triglycerides	0.015, 0.682		0.025, 0.529	
	**1983**	BMI	0.054, 0.213		**0.125, 0.006**	0.003
		Systolic blood pressure	0.046, 0.128		0.065, 0.138	
		Diastolic blood pressure	0.012, 0.748		0.038, 0.355	
		HDL-cholesterol	-0.052, 0.187		-0.049, 0.241	
		Triglycerides	0.002, 0.950		0.046, 0.263	
	**1986**	BMI	**0.080, 0.026**	0.032	0.016, 0.732	
		Systolic blood pressure	0.059, 0.200		0.053, 0.190	
		Diastolic blood pressure	**0.091, 0.034**	0.037	**0.077, 0.035**	0.003
		HDL-cholesterol	-0.055, 0.185		-0.020, 0.648	
		Triglycerides	0.027, 0.510		0.041, 0.343	
		Glucose	0.031, 0.458		**0.141, 0.001**	0.025
**Adulthood**	**2001**	BMI	0.070, 0.113		0.030, 0.532	
		Waist circumference	0.072, 0.090		0.067, 0.201	
		Systolic blood pressure	**0.093, 0.042**	0.038	**0.087, 0.047**	0.008
		Diastolic blood pressure	0.040, 0.387		0.037, 0.446	
		HDL-cholesterol	-0.051, 0.297		-0.083, 0.079	
		Triglycerides	0.040, 0.383		0.046, 0.344	
		Glucose	0.032, 0.479		0.034, 0.477	
	**2007**	BMI	0.076, 0.094		0.056, 0.200	
		Waist circumference	0.067, 0.161		**0.105, 0.040**	0.009
		Systolic blood pressure	0.067, 0.144		**0.085, 0.048**	0.007
		Diastolic blood pressure	**0.086, 0.048**	0.008	**0.083, 0.050**	0.007
		HDL-cholesterol	-0.031, 0.524		-0.051, 0.215	

		Triglycerides	0.043, 0.356		0.021, 0.666	
		Glucose	0.032, 0.480		0.026, 0.587	
	**2011**	BMI	**0.122, 0.009**	0.022	0.069, 0.150	
		Waist circumference	**0.092, 0.042**	0.016	0.013, 0.800	
		Systolic blood pressure	0.068, 0.197		**0.140, 0.008**	0.012
		Diastolic blood pressure	**0.102, 0.037**	0.028	**0.137, 0.008**	0.013
		HDL-cholesterol	-0.022, 0.662		-0.059, 0.264	
		Triglycerides	0.009, 0.856		0.020, 0.696	
		Glucose	0.012, 0.794		0.023, 0.646	

Oral infections/inflammations presented as number of decayed, missing, and filled teeth (DMFT) or percentage of sites (bleeding and probing pocket depth). The models are adjusted for age, sex, childhood BMI and family income. BMI and waist circumference are not adjusted for BMI. R^2 values are reported from corresponding unadjusted simple linear regressions with fitting models. Statistically significant values are bolded.

Supplementary figure. Frequencies of oral infections in children with or without MetS in adulthood.

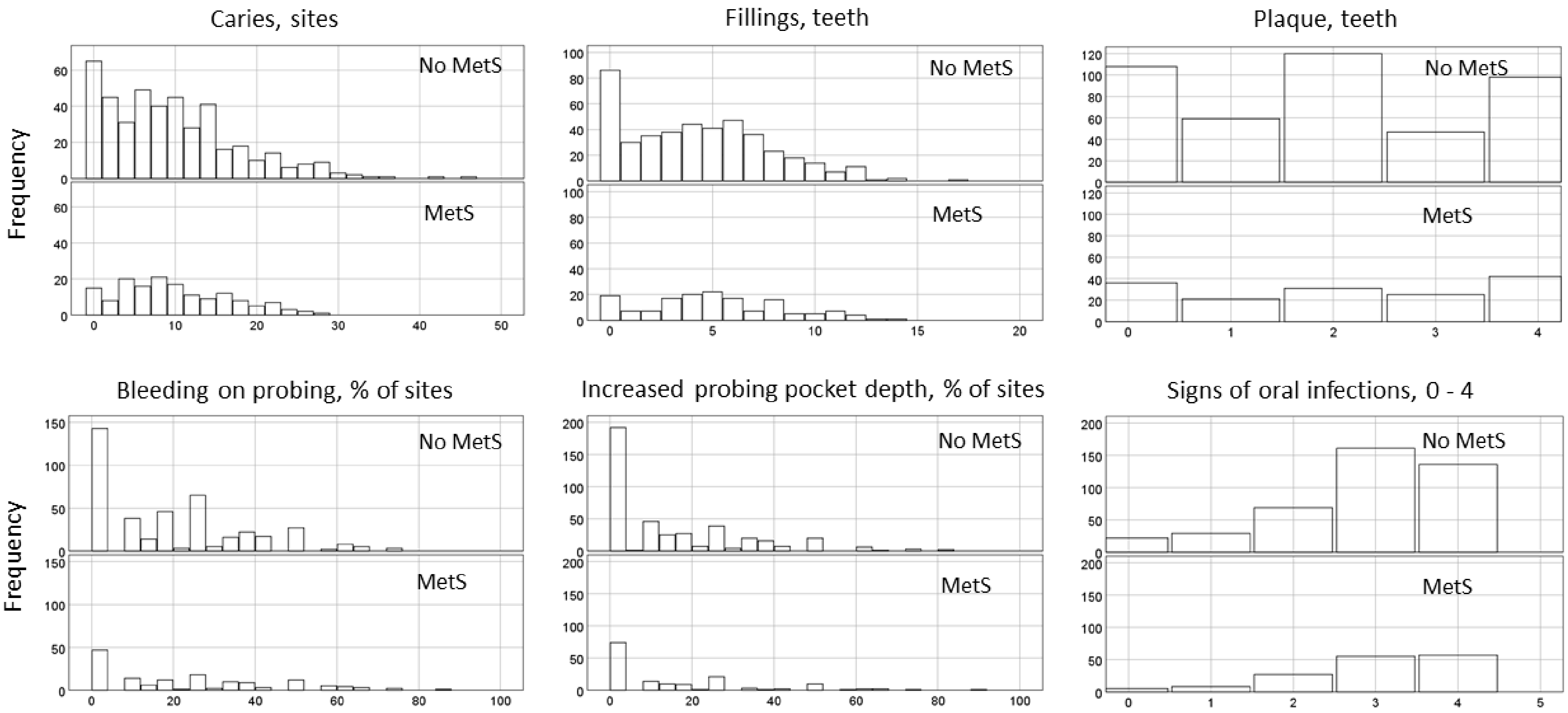

Supplement: DS_10.1177_0022034520929271 – Supplemental material for Childhood Oral Infections Associate with Adulthood Metabolic Syndrome: A Longitudinal Cohort Study [file DS_10.1177_0022034520929271.pdf]
